# Genetic variation of rs12918566 affects GRIN2A expression and is associated with spontaneous movement response during sevoflurane anesthesia induction

**DOI:** 10.1002/brb3.2165

**Published:** 2021-07-21

**Authors:** Ming‐Hua Chen, Chao Fang, Na‐Yiyuan Wu, Yu‐Hao Xia, You‐Jie Zeng, Wen Ouyang

**Affiliations:** ^1^ Department of Anesthesiology Third Xiangya Hospital of Central South University Changsha China; ^2^ Postdoctoral Research Station of Clinical Medicine Third Xiangya Hospital of Central South University Changsha China; ^3^ Hunan Cancer Hospital The Affiliated Cancer Hospital of Xiangya School of Medicine Central South University Changsha China

**Keywords:** expression, N‐methyl‐D‐aspartate, polymorphism, sevoflurane, spontaneous movement

## Abstract

N‐methyl‐D‐aspartate (NMDA) receptors mediate excitatory neurotransmission in the nervous system and are preferentially inhibited by general anesthetics such as sevoflurane. Spontaneous movement is a common complication during sevoflurane anesthesia induction and seriously affects operations. In this study, we investigated the relationship between NMDA polymorphisms and spontaneous movement during sevoflurane induction. This prospective clinical study enrolled 393 patients undergoing sevoflurane anesthesia as part of their surgical routine. In the GRIN1, GRIN2A, and GRIN2B genes, 13 polymorphisms that form a heteromeric complex as part of the NMDA receptor were selected using Haploview and genotyped using matrix‐assisted laser desorption ionization–time of flight mass spectrometry MassARRAY. Both RNAfold and Genotype‐Tissue Expression portals were used to identify gene expression profiles. Our data showed that 35.8% of subjects exhibited spontaneous movement. The GRIN2A rs12918566 polymorphism was associated with spontaneous movement during sevoflurane induction. A logistic regression analysis of additive, dominant, and recessive models indicated a significant association (odds ratio [OR] (95% confidence limit [CI]): 0.58 (0.42–0.80), *p* = .00086; OR (95% CI): 0.51 (0.31–0.84), *p* = .0075, and OR (95% CI): 0.47 (0.27–0.81), *p* = .0060, respectively). After false discovery rate (FDR) correction, the additive model was still significant with a *P*_FDR_ =0.010. Bioinformatics demonstrated that the rs12918566 genomic variation affected GRIN2A expression in brain tissue. We also revealed that GRIN2A rs12918566 was significantly associated with spontaneous movement during sevoflurane induction. We believe the NMDA receptor plays an important role in regulating the anesthetic effects of sevoflurane.

## Graphical abstract

The present study revealed that GRIN2A rs12918566 was significantly correlated with spontaneous movement during sevoflurane anesthesia induction, and this locus can significantly affect the expression of GRIN2A. Suggested NMDA receptor plays an important role in regulating anesthetic effect of sevoflurane.

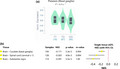

## 1 INTRODUCTION

Sevoflurane is a volatile anesthetic approved by the Food and Drug Administration for the induction and maintenance of general anesthesia and is one of the most commonly used volatile anesthetics in pediatric, adult, and outpatient surgical patients (Brioni et al., 2017; De Hert & Moerman, 2015). Sevoflurane provides hypnosis, amnesia, analgesia, immobility, and autonomic blockade during surgical and procedural interventions. It is commonly accepted that amnesia, sedation/hypnosis, and immobility are the three vital anesthesia requisites (Constant & Sabourdin, 2015). However, like other volatile anesthetics, brief spontaneous movements often occur during sevoflurane anesthesia induction and recovery, with some patients experiencing epileptiform electroencephalogram changes (Jantti & Sonkajarvi, 2004; Vlajkovic & Sindjelic, 2007; Yli‐Hankala et al., 1999). Studies have reported that spontaneous movement incidences in children receiving sevoflurane may be as high as 60%–91% (Akeson & Didriksson, 2004; Chawathe et al., 2005; Constant et al., 1999). An ideal anesthetic agent is characterized by anesthetic and analgesic actions, no adverse effects on the respiratory and circulatory system, and no irritation to the skin and mucous membranes. Severe spontaneous movements during anesthesia may lead to patients falling out of bed or surgical complications and seriously affect patient safety and surgical progress (Borgeat et al., 1991; Chan et al., 1996; Nagata et al., 2004; Reynolds & Koh, 1993).

Studies have shown the spinal cord is the primary site affected by volatile anesthetics, often causing immobility, whereas sedation (hypnosis) and amnesia involve supraspinal mechanisms (Jinks et al., 2008). However, the molecular mechanism underpinning spontaneous movement during sevoflurane induction is unclear. Scientists have discovered that sevoflurane alters postsynaptic currents by acting on ion channel receptors on the striatal synapse, generating synaptic excitation and spontaneous movement (Oose et al., 2012).

Studies on the isolated spinal cord have suggested that actions involving excitatory α‐amino‐3‐hydroxy‐5‐methyl‐4‐isoxazole‐propionic acid and N‐methyl‐D‐aspartate (NMDA) receptors but not inhibitory (glycine and γ‐aminobutyric acid type A [GABA_A_]) transmission may produce immobility (Sonner et al., 2003). NMDA receptors are composed of a major class of excitatory ligand‐gated ion channels in the nervous system. Their transient activation leads to long‐term modification of synaptic connections and refinement of neuronal circuits, which are involved in learning and memory formation (Irifune et al., 2007). Ishizaki *et al*. reported a maximal 30% decrease in isoflurane minimum alveolar concentration (MAC) in rats given intrathecal NMDA antagonist bolus doses (Ishizaki et al., 1996). A study investigating antagonized NMDA receptors indicated that spinal cord NMDA receptors might mediate a portion of the immobilizing effects of isoflurane (Stabernack et al., 2003).

Molecular studies have revealed that NMDA receptors exist as heteromeric complexes composed of GRIN1, GRIN2A‐D, and GRIN3A‐B subunits (Paoletti et al., 2013). GRIN1 is the essential NMDA receptor channel‐forming subunit that contains a glycine binding site. The GRIN2 subunit contains a glutamate binding site with affinities for agonists and antagonists. GRIN2A/Bs are abundant in the central nervous system (Nishikawa, 2011; Teng et al., 2010). In contrast, NMDA receptor sensitivity to anesthetics does not appear to depend on GRIN3 subunits (Petrenko et al., 2014). In a *Xenopus laevis* model expressing human GRIN1/GRIN2B receptor channels, equal concentrations of inhaled anesthetics inhibited NMDA receptors to varying degrees (the inhibitory effects of sevoflurane on GRIN1/GRIN2B expression were significantly higher than isoflurane: 14%–28%), and this result may be useful for defining the role of NMDA in producing general anesthetics (Solt et al., 2006). From this literature, GRIN1 and GRIN2A/B subunits are more likely to contribute to the effects of inhalation anesthesia.

Therefore, we hypothesized that NMDA polymorphisms might be associated with spontaneous movement responses induced by sevoflurane. Our aim was to determine associations between NMDA (GRIN1, GRIN2A, and GRIN2B), tag‐SNPs (tag‐single‐nucleotide polymorphisms), and spontaneous movement responses after sevoflurane induction.

## 2 METHODS

### 2.1 Patient recruitment

This prospective clinical study included 393 women who underwent hysteroscopy at the Third Xiangya Hospital of Central South University, Changsha, Hunan, China, between June and October 2018. All participants provided signed informed consent.

The study was approved by the Ethics Committee of The Third Xiangya Hospital of Central South University (project number: R18018) and the Chinese Clinical Trial Registry (registration number: ChiCTR1800017368).

Inclusion criteria were: (1) age ˃ 18 years, (2) American Society of Anesthesiologists classification (I–II), and (3) patients undergoing general anesthesia for hysteroscopy. Exclusion criteria were: pregnancy, fever, water and electrolyte disorder, hyperthyroidism, severe anemia, serious history of cardiopulmonary disease, and abnormal liver and kidney function.

### 2.2 Treatment

Patients received the following treatment before general anesthesia: fasted for 8 hr and received no liquids for 2 hr prior to anesthesia. An intravenous infusion channel was established before anesthesia, and a multi‐function monitor assessed blood pressure, heart rate, and electrocardiogram output. Bispectral index (BIS) was used to monitor anesthesia depth. Patients inhaled 3% sevoflurane (5 L/min) for 4 min using a pressurized sealing mask. The sevoflurane was then replaced with air and administered at 5 L/min for 2 min and then propofol for routine anesthesia. Throughout anesthesia, the airway was opened and ventilation assisted based on end‐of‐breath carbon dioxide partial pressure (PaCO_2_) (maintained at 35–45 mm Hg). Heart rate (HR) and blood pressure were monitored. When the HR was <50 beats/min, patients were given atropine (0.3 mg–0.5 mg). When the mean arterial pressure (MAP) was <30% of baseline, 5 mg ephedrine was given, and 0.25 mg nitroglycerin was given when the MAP was >30% of baseline.

Modified observer's assessment of alertness/sedation scoring was performed every 30 s; (5, readily responds to the name spoken in a normal tone; 4, lethargic response to the name spoken in a normal tone; 3, responds only after the name is loudly and/or repeatedly called; 2, responds only after mild prodding or shaking; 1, responds only after a painful trapezius squeeze; 0, no response after a painful trapezius squeeze). Patients were deemed unconscious when the score was <2. Spontaneous movement criteria were also applied: grade 0: no movement, quiet, cooperative; grade I: mild movement, limited to hands or feet, no rotation in arms or legs; grade II: moderate/mild movement, arm or leg flexion or extension; Grade III: strenuous/moderate exercise, plus internal or external rotation of shoulders or hips and knee, severely struggling and required an external force to stop (Borgeat et al., 1991). In our study, a 6‐min evaluation period was observed after sevoflurane administration, and the occurrence of any movement in grade I, II, and III was defined as a spontaneous movement. The BIS value, end‐tidal anesthetic concentration (ETAC), and MAC were also recorded when patient activity appeared.

### 2.3 Sample collection, DNA extraction, single‐nucleotide polymorphism selection, and genotyping

We collected 2 ml venous blood in ethylenediaminetetraacetic acid anticoagulant tubes. DNA was extracted using a HiPure Blood DNA Mini Kit (Magen, China) according to the manufacturer's protocols. Extracted DNA was stored at −20℃.

Thirteen tagSNPs from GRIN1, GRIN2A, and GRIN2B were selected. All SNPs met the following criteria: (1) a minor allele frequency (MAF) > 5% in the Chinese Han population, (2) SNPs were selected by Haploview (version 4.2) using pairwise linkage disequilibrium with default settings (the Hardy–Weinberg *P* value cutoff was 0.05 and r^2^ > 0.8).

Assay Designer software (version 3.1) was used for the selected SNP primer design. Polymorphisms were detected using the Sequenom MassARRAY Genotype Platform (Sequenom, San Diego, California, USA) using an allele‐specific matrix‐assisted laser desorption ionization–time of flight mass spectrometry method.

### 2.4 Effects of genetic variation on gene expression

The RNAfold web server (http://rna.tbi.univie.ac.at/cgi‐bin/RNAWebSuite/RNAfold.cgi) was used to predict potential functions from genetic variations. To evaluate such relationships, expression quantitative trait loci (eQTL) were analyzed using the Genotype‐Tissue Expression (GTEx) portal database (https://www.gtexportal.org/home/).

### 2.5 Statistical analyses

Statistical analyses were performed using SPSS 18.0 for Windows (IBM, Inc., Chicago, IL, USA) and PLINK 1.07. Counting data were presented as the mean ± standard deviation. Differences between the two groups were compared using the independent sample *T* test, while the one‐way analysis of variance was performed to compare more than two groups. *p* <.05 indicated statistical significance. Associations between genotypes and spontaneous movement were performed using a logistic regression model, and corrections for potential confounders were performed by adjusting for covariates, for example, age, ETAC.

## 3 RESULTS

### 3.1 Single‐nucleotide polymorphism selection and genotyping

We selected 13 tagSNPs in GRIN1 and GRIN2A/B for genotyping (Table 1). Our MAF values were consistent with reference values from the Ensembl database (http://asia.ensembl.org/index.html), which verified the accuracy of our genotyping method. GRIN2B rs71379053 genotyping was unsuccessful because several homologous sequences were observed upstream and downstream of the locus. The call rate of SNPs was >95%, and SNPs were in accordance with Hardy–Weinberg equilibrium, except GRIN2B rs1019385 (*P*_hw_ <0.05). For these reasons, GRIN2B rs71379053, and rs1019385 were excluded, and 11 tagSNPs were included for subsequent analysis (Table 1). Specific genotyping primer sequences are shown in Table 2.

**TABLE 1:** Characters of SNPs genotyped in this study

Gene	SNP	Position	Category	Allele	Call rate (%)	MAF (%)		*P*_HW_
						a	b	
GRIN1	rs2301363	chr9:137139979	Intron Variant	C > T	100.00	24.8	24.05	0.78
	rs79901440	chr9:137146368	Intron variant	C > T	97.96	5.2	5.33	0.61
	rs28681971	chr9:137152639	Intron variant	C > T	100.00	8.1	8.52	0.34
GRIN2A	rs837701	chr16:10018707	Intron variant	T > C	98.47	47.6	45.99	0.41
	rs9788939	chr16:10012049	Intron variant	T > A	99.49	38.1	34.53	0.65
	rs12918566	chr16:10109692	Intron variant	T > G	99.75	45.2	43.24	0.41
	rs71379053	chr16:9785189	Intron variant	T > C	98.73	29.0	NA	1
GRIN2B	rs1019385	chr12:13981909	Intron variant	C > A	98.47	42.4	43.02	<0.05
	rs1806201	chr12:13564574	Intron variant	G > A	100.00	44.8	46.06	1
	rs2160734	chr12:13831415	Intron variant	C > T	100.00	21.4	29.77	1
	rs2284411	chr12:13713238	Intron variant	C > T	100.00	22.4	22.26	0.06
	rs890	chr12:13562374	3′ UTR variant	A > C	100.00	19.5	23.66	0.78
	rs1072388	chr12:13785842	Intron variant	G > A	100.00	10.0	10.94	0.11

a: 1,000 Genomes of Southern Han Chinese.

b: in this study.

**TABLE 2:** Primers of all the selected SNPs

SNPs	2nd‐PCRP	1st‐PCRP
rs2301363	ACGTTGGATGCACTAGGAACCAAACACCAG	ACGTTGGATGACGTCTACACACACCAGTCA
rs79901440	ACGTTGGATGCTTTGCTGAGCCATGAGGTG	ACGTTGGATGCTGTACACGACCCCCACATA
rs28681971	ACGTTGGATGTGTCTGGAAACGTGACCTTG	ACGTTGGATGTTTTTTCTCGGCCTGTGAGC
rs837701	ACGTTGGATGCATCTTCATGGTGAAAGCGG	ACGTTGGATGAAACTCTATCCAGGGAGGTG
rs9788939	ACGTTGGATGTCGCTCCCACTCAAGAAAAC	ACGTTGGATGTGTGAGGTGTGATGCACTAA
rs12918566	ACGTTGGATGATGAGGTCTCCATCCCCTTG	ACGTTGGATGGAAGAATGAATAAGACCTACC
rs71379053	ACGTTGGATGGGCTGCATAGTATTCCATGT	ACGTTGGATGGCAGCACTATTCACAATAGC
rs1019385	ACGTTGGATGAGTATCTTCCCCAGCTTCGT	ACGTTGGATGTCACACTCAAAAACGTGCCG
rs1806201	ACGTTGGATGAGCGCCAGTCTGTAATGAAC	ACGTTGGATGTTCACACCAGACAGGTTAGC
rs2160734	ACGTTGGATGCTGTGACTCCATGCCTGTTG	ACGTTGGATGCCAAATGACAATGACAGAGC
rs2284411	ACGTTGGATGTAGGGCTAATAGGAATGGAG	ACGTTGGATGACATGACTTTTTTCCCCTAC
rs1072388	ACGTTGGATGACTGGCTCCTAGAGTCTTAG	ACGTTGGATGGGCAACTGAGACTCAAAATG
rs890	ACGTTGGATGTCCTCTTTGAGTGAAGCTGG	ACGTTGGATGGCAGTGTGCTAAATGGTCTC

Note

PCRP: polymerase chain reaction primer.

### 3.2 Study population clinical characteristics

From sedation scores, all patients reached a hypnotic (unconscious) state within 4 min, except for one case who lost consciousness at 280 s (Table 3).

**TABLE 3:** Clinical features of the study cohort

	Patients (*n* = 393)		*p* value
Characteristics	Spontaneous movement		
	Y−1	N−2	
*n*	130	263	
Age	31.92 ± 4.96	32.84 ± 5.49	0.10
BMI	21.49 ± 3.04	21.53 ± 2.71	0.889
˂18.5	18	31	
18.5–25	96	206	
≥25	16	26	0.107
ASA			
I	26	73	
II	61	114	
III	32	65	0.334
HB (g/L)	104.42 ± 54.46	95.78 ± 57.80	0.156
T‐_BIS(min)_	232.46 ± 48.76	227.13 ± 47.39	0.368
T‐_unconsciousness_	118.15 ± 29.92	117.22 ± 28.21	0.762
BIS‐_min_	22.87 ± 15.72	23.07 ± 17.07	0.909
MAC‐_BIS(min)_	0.61 ± 0.42	0.62 ± 0.43	0.88
ETAC‐_BIS(min)_	232.45 ± 48.76	227.13 ± 47.39	0.368

Abbreviations: ASA, American Society of Anesthesiologists; BIS, bispectral index; BMI, body mass index; ETAC, end‐tidal anesthetic concentration; HB, hemoglobin; MAC, minimum alveolar concentration; min: minimum.

Of the 363 patients included, 130 (35.8%) developed spontaneous movement reactions after receiving the prescribed sevoflurane dose, while the remaining 263 showed no spontaneous movement. Patient clinical characteristics are summarized in Table 3. Both groups (130 patients; spontaneous movement group and 263 patients; no spontaneous movement group) were young with average ages of 31.92 ± 4.96 and 32.84 ± 5.49 years, respectively. We observed no significant differences in clinical characteristics between groups.

### 3.3 GRIN2A rs12918566 was significantly associated with spontaneous movement responses after sevoflurane

Associations between candidate gene polymorphisms and spontaneous movement responses were analyzed by Plink using a logistic model. Age and ETAC covariates were adjusted as previous studies reported they were associated with volatile anesthetic drug responses (Dwyer et al., 1990; Moshchev & Lubnin, 2014; Sakai et al., 2005). The additive, dominant, and recessive models indicated significant associations of GRIN2A rs12918566 and spontaneous movement (odds ratio (OR) (95% confidence limit (CI)): 0.58 (0.42–0.80), *p* =.00086; OR (95% CI): 0.51 (0.31–0.84), *p* =.0075; OR (95% CI): 0.47 (0.27–0.81), *p* =.0060, receptively). After false discovery rate (FDR) correction, we still observed significance for the additive model; *P*_FDR_ =0.010 (Table 4). Other association analysis data are presented in Table 5.

**TABLE 4:** Association between GRIN2A rs12918566 and spontaneous movement response during sevoflurane induction

Gene	SNP	Genotype			Genetic model	OR (95%CI)	*p* ^a^	*p_FDR_*
			*N*	Y				
GRIN2A	rs12918566	TT	36	33	Additive	0.58 (0.42–0.80)	0.000858	0.0103
		TG	132	69	Dominant	0.51 (0.31–0.84)	0.0075	0.0902
		GG	94	28	Recessive	0.47 (0.27–0.81)	0.00605	0.072

^a^ Adjusted by age and ETAC.

**TABLE 5:** Association of other SNPs and spontaneous movement response in sevoflurane induction

Gene	SNPs	Genotype	Spontaneous movement		OR (95%CI)	*p*
			Y	*N*		
GRIN1	rs2301363	CC	79	149	1.27 (0.89–1.81)	0.187
		CT	47	94		
		TT	4	20		
	rs79901440	CC	110	234	0.81 (0.42–1.60)	0.552
		CT	15	26		
						
	rs28681971	TT	106	221	0.80 (0.47–1.38)	0.43
		TC+CC#	23	42		
						
GRIN2A	rs837701	CC	32	54	0.87 (0.65–1.17)	0.358
		TC	58	126		
		TT	36	81		
	rs9788939	AA	18	26	1.02 (0.74–1.40)	0.901
		TA	53	129		
		TT	59	106		
GRIN2B	rs1019385	CC	18	40	1.03 (0.74–1.42)	0.868
		CA	74	143		
		AA	37	75		
	rs1806201	GG	24	59	1.20 (0.89–1.62)	0.238
		GA	64	132		
		AA	42	72		
	rs2160734	TT	12	23	1.13 (0.89–1.81)	0.187
		TC	49	115		
		CC	69	125		
	rs2284411	TT	9	17	1.03 (0.73–1.45)	0.877
		TC	39	84		
		CC	82	162		
	rs890	CC	3	20	1.23 (0.86–1.76)	0.248
		CA	49	91		
		AA	78	152		
	rs1072388	GG	104	211	1.02 (0.65–1.62)	0.917
		GA	24	46		
		AA	2	6		

### 3.4 GRIN2A rs12918566 genomic variation is associated with GRIN2A expression

**FIGURE 1:**
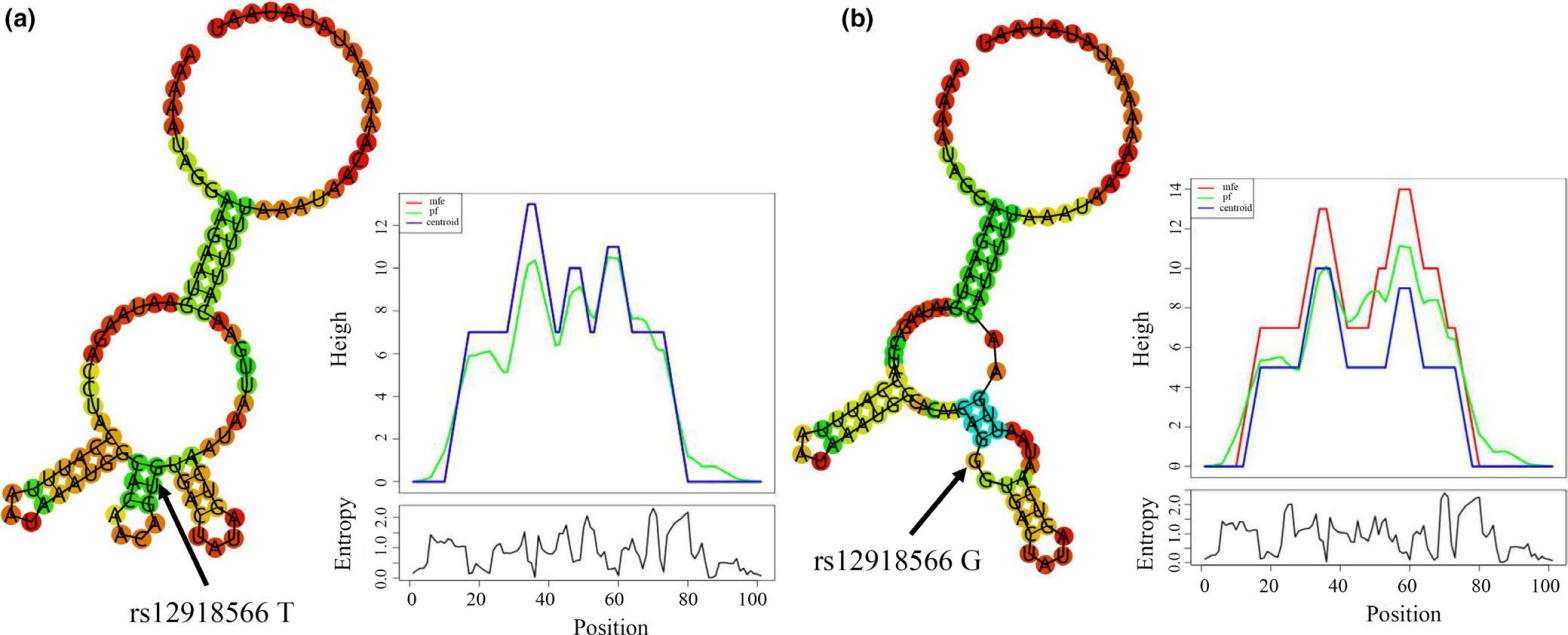
The secondary structure of GRIN2A mRNA contains different rs12918566 alleles. (a) GRIN2A structure containing the rs12918566 T allele. The minimum free energy (MFE) of the structure is −10.10 kcal/mol. (b) GRIN2A structure containing the rs12918566 G allele. The MFE of the structure is −8.60 kcal/mol. ^#^Secondary structures and MFEs were predicted using the 50 base pair sequences upstream and downstream of the rs12918566 mutation site

**FIGURE 2:**
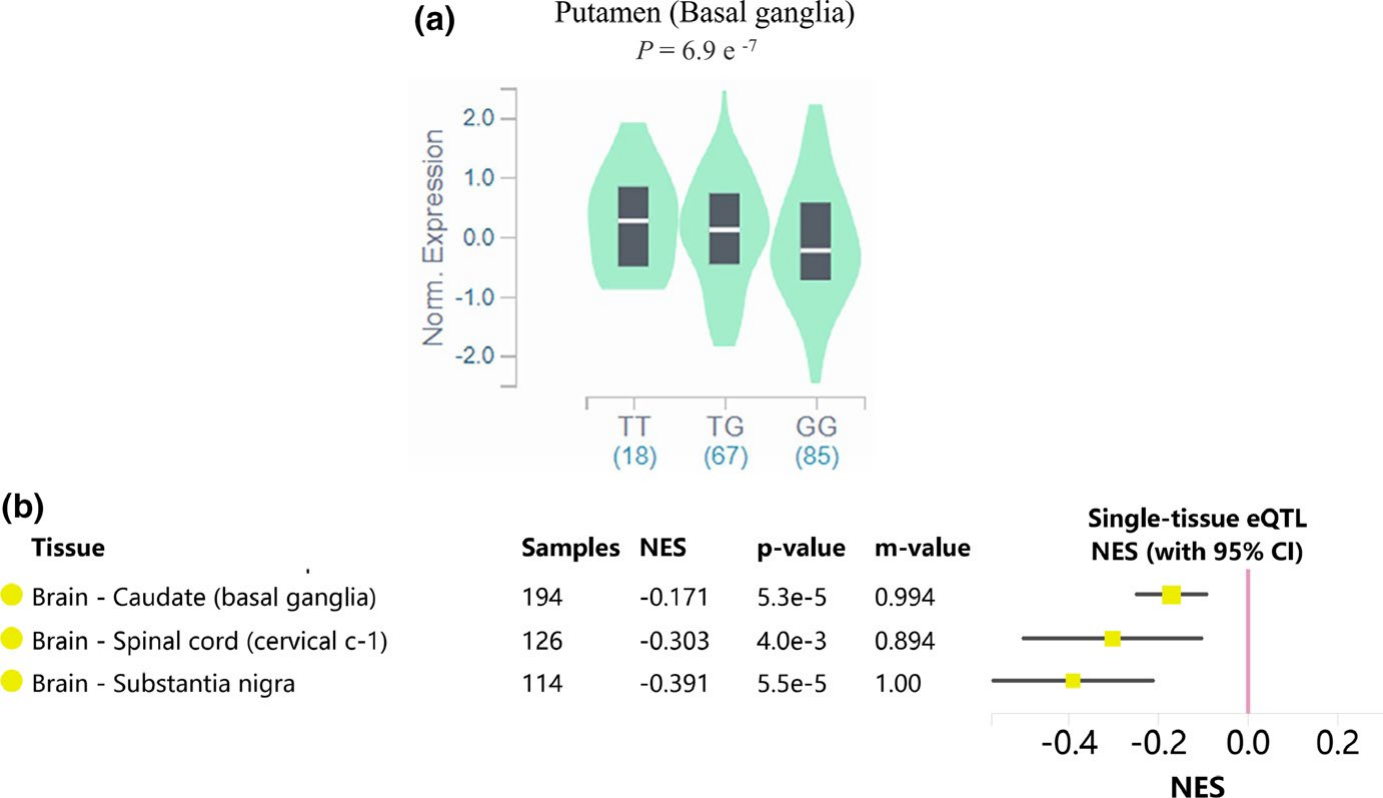
Single tissue expression quantitative trait locus analysis for GRIN2A rs12918566. (a) GRIN2A mRNA expression in basal ganglia between different rs12918566 genotypes. (b) GRIN2A rs12918566 polymorphism was significantly associated with GRIN2A expression in basal ganglia, spinal cord, and substantia nigra

Bioinformatics analysis indicated that the GRIN2A rs12918566 polymorphism significantly altered GRIN2A mRNA secondary structure, and the free energy of the mutant (G allele) increased (−8.60 kcal/mol) when compared with the wild type (T allele, −10.10 kcal/mol) (Figure 1). Furthermore, eQTL analysis in 170 tissues showed that the TG and GG genotypes of rs12918566 displayed lower GRIN2A expression in brain basal ganglia when compared with TT individuals (Figure 2a). This association also existed in other brain sections and the spinal cord (Figure 2b).

## 4 DISCUSSION

Sevoflurane has been clinically used for more than 20 years; however, its precise induction mechanism and general anesthesia maintenance characteristics remain unclear. Glutamate is the most concentrated excitatory neurotransmitter in the brain and spinal cord and mediates postsynaptic membrane effects mainly via ionotropic and metabotropic receptors (Reiner & Levitz, 2018). As a class of ionotropic receptors, NMDA receptors have a wide range of functions in the basic synaptic transmission, neuroplasticity, and neurodegenerative diseases of the central nervous system; the domain of the GRIN2 subunit contains a glutamate binding site (Daw et al., 1993; Fang et al., 2014; Lee et al., 2010). This study was the first to investigate the effects of NMDA receptor genetic polymorphisms on spontaneous movement response during sevoflurane anesthesia. We revealed that GRIN2A rs12918566 was significantly associated with sevoflurane‐induced spontaneous movement during anesthesia induction.

Transient NMDA receptor activation induces the long‐term modification of synaptic connections and the refinement of neuronal circuits and may be the basis of learning and memory (Jeon et al., 2008). In a rat model, intrathecal administration of the NMDA receptor antagonist, MK801, reduced the MAC value of isoflurane in a dose‐dependent manner, but this effect was reversed by NMDA receptor agonist administration (Ishizaki et al., 1996, 1997; Stabernack et al., 2003). The MAC value for an inhaled anesthetic is similar to the ED50 (median effective dose) for an intravenous anesthetic; that is, the lowest alveolar inhalation anesthetic concentration at 1 atmosphere when 50% of patients no longer experience body movement during surgical procedures(Hodgson & Liu, 2001). Thus, NMDA appears to have important roles in regulating anesthetic capacity (Rudolph & Antkowiak, 2004).

The GRIN2 (A–D) subunits are the main determinants of receptor functional heterogeneity and exhibit strikingly different spatiotemporal expression (Monyer et al., 1994). The GRIN2A gene encodes the NMDA receptor GRIN2A subunit, which is located on chromosome 16p13.2 and consists of 12 exons and 13 introns (Liu & Zhao, 2013). A patch‐clamp study reported that the inhibitory effects of xenon on NMDA receptor‐mediated currents were weakened when transmembrane segment four of GRIN2A or transmembrane segment three of GRIN1 were mutated (Dickinson et al., 2007). GRIN2A is present throughout the dorsal horn except in lamina II at the spinal cord level, while GRIN2B does not appear to exist in lamina II and is restricted to certain areas in the superficial dorsal horn (Boyce et al., 1999; Watanabe et al., 1992). Physiologically, this suggests GRINA2A may play a potential role in immobilization effects because volatile anesthetic‐induced immobility results from actions on the spinal cord (Antognini et al., 2002). GRINA2A subunit knockout mice were resistant to the hypnotic effects (loss of righting reflex) produced by ketamine (Sato et al., 2004). Mutation of the transmembrane segment of the GRIN2A subunit (A825W) significantly reduced recombinant NMDA receptor sensitivity to halothane, isoflurane, cyclopropane, and xenon (Ogata et al., 2006). Also, the release of GABA stimulated by NMDA in striatum slices was significantly decreased in GRIN2A knockout mice (Miyamoto et al., 2001). NMDA receptors are possible targets of inhaled anesthetic components and are involved in the braking of general anesthesia and loss of consciousness (Petrenko et al., 2014).

Our results suggest NMDA is involved in regulating spontaneous movement during sevoflurane induction. Individuals with GRIN2A rs12918566 T alleles were not sensitive to sevoflurane and were more prone to experiencing spontaneous movement during sevoflurane administration.

From in silico predictions using RNAfold, we observed a significant difference in the free energy of the secondary structure, and GTEx database analysis showed that the rs12918566 locus was highly associated with GRIN2A expression in the brain. We speculate the rs12918566 polymorphism regulates GRIN2A gene expression by altering GRIN2A mRNA secondary structure and functioning in the patient's responses to sevoflurane.

In summary, we showed the GRIN2A rs12918566 locus was significantly associated with spontaneous movement during sevoflurane induction in Chinese Han women. Therefore, we speculate the GRIN2A subunit may have important roles in volatile anesthesia.

## CONFLICT OF INTEREST

The authors disclose no conflict.

## AUTHOR CONTRIBUTIONS

CMH, CF, and NYW analyzed the data and wrote this manuscript. CF, MHC, YHX, and YJZ performed experiments and visualization. NYW and MHC aided in processing the data. YHX and YJZ aided in the collection of the materials. NYW helped to revise this manuscript. CF, MHC, and WOY conceived and designed the experiments.

### PEER REVIEW

The peer review history for this article is available at https://publons.com/publon/10.1002/brb3.2165.
